# A Pseudovirus-Based Neutralization Assay for SARS-CoV-2 Variants: A Rapid, Cost-Effective, BSL-2–Based High-Throughput Assay Useful for Vaccine Immunogenicity Evaluation

**DOI:** 10.3390/microorganisms12030501

**Published:** 2024-02-29

**Authors:** Zhaohui Cai, Raj Kalkeri, Mingzhu Zhu, Shane Cloney-Clark, Benjamin Haner, Mi Wang, Bahar Osman, Dominic Dent, Sheau-Line Feng, Zach Longacre, Greg Glenn, Joyce S. Plested

**Affiliations:** 1 Clinical Immunology, Novavax, Gaithersburg, MD 20878, USA; zcai@novavax.com (Z.C.); rkalkeri@novavax.com (R.K.); mzhu@novavax.com (M.Z.); wcloneyclark@novavax.com (S.C.-C.); bhaner@novavax.com (B.H.); mwang@novavax.com (M.W.); bosman@novavax.com (B.O.); ddent@novavax.com (D.D.); sfeng@novavax.com (S.-L.F.); 2 Discovery, Novavax, Gaithersburg, MD 20878, USA; zlongacre@novavax.com (Z.L.); gglenn@novavax.com (G.G.)

**Keywords:** COVID-19, pseudovirus-based neutralization assays, immunogenicity, SARS-CoV-2, assay validation, neutralizing antibody titers, correlate of protection, XBB.1.5, XBB.1.16, XBB.2.3

## Abstract

Neutralizing antibody responses from COVID-19 vaccines are pivotal in conferring protection against severe acute respiratory syndrome coronavirus 2 (SARS-CoV-2). Effective COVID-19 vaccines and assays measuring neutralizing antibodies against emerging variants (i.e., XBB.1.5, XBB.1.16, and XBB.2.3) are needed. The use of biosafety level (BSL)-3 laboratories for live virus assays results in higher costs and a longer turnaround time; therefore, a BSL-2–based pseudovirus neutralization assay (PNT) was developed. The pseudoviruses were produced by cotransfecting cells with plasmids encoding a lentiviral backbone-expressing luciferase reporter; non-surface proteins for lentiviral production; and ancestral or Omicron (BA.1 and BA.5) SARS-CoV-2 spike (S) proteins. The PNT was developed and optimized in dose and kinetics experiments. The representative serum samples (COVID-19–convalescent or NVX-CoV2373–vaccinated participants enrolled in the 2019nCoV-101 trial) demonstrated a wide dynamic range. The neutralization data showed robust correlation with validated anti-recombinant spike IgG levels and angiotensin-converting enzyme 2 inhibition titers (ancestral). This assay is suitable for measurement of the neutralization ability in clinical samples from individuals infected with SARS-CoV-2 or immunized with a COVID-19 vaccine. The results suggest that this PNT provides a lower cost, high-throughput, rapid turnaround alternative to BSL-3–based microneutralization assays and enables the discovery and development of effective vaccines against emerging variants.

## 1. Introduction

The SARS-CoV-2 virus, responsible for COVID-19, elicits neutralizing antibodies as part of the immune response to counter viral infection [1]. Neutralizing antibodies help mediate the humoral immune response to viral infection, playing a key role in early viral clearance [2]. In addition to being a critical component of protection against early anti-viral responses, it was reported that neutralizing antibodies can be measured as a potential correlate of infection-driven protection or a correlate of protection induced by vaccination [3]. Specifically, neutralizing antibody titers had a significant inverse correlation with the risk of symptomatic COVID-19 in an exploratory analysis of the PREVENT-19 trial investigating the NVX-CoV2373 COVID-19 vaccine.

New SARS-CoV-2 variants continue to emerge, some of which are even more transmissible and virulent than the ancestral strain. The latest variants of concern/interest prioritized by the World Health Organization include the Omicron XBB.1.5, XBB.1.16, and XBB.2.3 strains [4]. Notably, emerging variants have several mutations in the spike (S) protein to differentiate them from the ancestral and early variant strains [5], which may support their ability to evade the immune systems of those previously infected or vaccinated [6]. With the breadth of these mutations and the occurrence of breakthrough infections, there is a continuous need to assess the ability of vaccines to counter these new variants [7]. The current standards for the testing of SARS-CoV-2 clinical trial samples are expensive, laborious, and time-consuming, due to the need of a biosafety level (BSL)-3 facility for live virus microneutralization assays [8]. The BSL-3 regulations require more stringent laboratory safety precautions related to the handling of a live virus compared with routine BSL-2 laboratories [9]. Additionally, differences in the epitopes and replication kinetics among strains may require adaptations (e.g., different cell lines and/or incubation periods) to the live virus assay, which will require optimization and retraining of personnel, prolonging the period of time before the modified assay may be used [10].

Pseudoviruses are artificially engineered viruses that lack some of the genetic sequences of the original virus [8,11]; therefore, they can be handled in BSL-2 (instead of BSL-3) conditions [8,12]. A critical advantage to the pseudovirus system is the adaptability of the assay to accommodate the investigation of viral variants. Assays with a quick turnaround are of immediate importance for assessing the immunogenicity of vaccines that express variant S proteins to help provide the information needed when considering the implementation of new vaccines based on emerging variants.

This study was aimed at developing a novel pseudovirus-based neutralization assay that could be used for both the ancestral and variant strains of SARS-CoV-2. The pseudovirus neutralization assay (PNT) described here can detect and quantitate neutralizing antibodies against the SARS-CoV-2 ancestral strain and variants, which can further help in evaluating vaccine immunogenicity.

## 2. Materials and Methods

### 2.1. Cell Lines

Human embryonic kidney cells (HEK293T; ATCC CRL-3216; Manassas, VA, USA) and African Green Monkey kidney cells (Vero-E6; ATCC CRL-1586; Manassas, VA, USA) were cultured in Dulbecco’s Modified Eagle Medium (DMEM; Cat#11960, Gibco, Grand Island, NY, USA) with 10% fetal bovine serum (FBS; Cat#10082, Gibco, Grand Island, NY, USA), 2 mM *L*-glutamate, 100 U/mL penicillin, and 100 µg/mL streptomycin. Human lung carcinoma cells overexpressing human angiotensin-converting enzyme 2 (hACE2) and human transmembrane serine protease 2 (TMPRSS2; A549-hACE2-TMPRSS2, InvivoGen, San Diego, CA, USA) were cultured in the same medium as the HEK293T and VeroE6 cells, plus 0.5 µg/mL puromycin and 300 µg/mL hygromycin. Human embryonic kidney cells overexpressing hACE2 (293T/hACE2, Creative Biogene, Shirley, NY, USA) were cultured in the same medium as the HEK293T and VeroE6 cells, plus 1 µg/mL puromycin. The cell lines were authenticated at the source, as noted in the Certificate of Analysis provided by the vendor. Cell culture passage numbers less than 35 were used in the assays to limit any cell passage–associated issues/variability in the assays. Master Cell Bank mycoplasma testing was conducted to rule out any mycoplasma contamination.

### 2.2. Pseudoviruses

The HEK293T cells in T175 175 cm^2^ flasks (Thermo Fisher Scientific, Waltham, MA, USA) were cultured in DMEM with 10% FBS overnight before cotransfecting them with plasmids (adapted from Crawford 2020 [13]) encoding (1) lentiviral backbone-expressing luciferase/ZsGreen reporter, (2) lentiviral helper plasmids, and (3) SARS-CoV-2 S protein (a codon-optimized sequence from the ancestral strain or Omicron variants) under the control of a CMV promoter (synthesized by GenScript, Piscataway, NJ, USA). A cationic lipid-based/polymer-based transfection method (Lipofectamine^TM^ 3000 [Thermo Fisher] or jetOPTIMUS^®^ [Polyplus, Illkirch-Graffenstaden, France]) was used, according to the manufacturer’s protocol. The cell culture supernatants were collected 48 h post transfection, centrifuged at 1000 rpm for 10 min, filtered using a 0.45-µM filter, and used as a source of the pseudovirus. The commercial pseudoviruses for the Omicron strains XBB.1.5, XBB.1.16, and XBB.2.3 were obtained from eENZYME (Gaithersburg, MD, USA). To determine the virus titer, various dilutions of the cell culture supernatants were used to infect the 293T/hACE2 cells in 96-well white cell culture plates, followed by the addition of luciferase reagent (Bright-Glo^TM^ Luciferase Assay System, E2650, Promega, Madison, WI, USA) for the measurement of the luminescence levels 48 h or 72 h post infection. To allow for consistency, the pseudovirus batches were evaluated for infectivity prior to using them for the sample testing.

### 2.3. Assay Procedure

**Figure 1:**
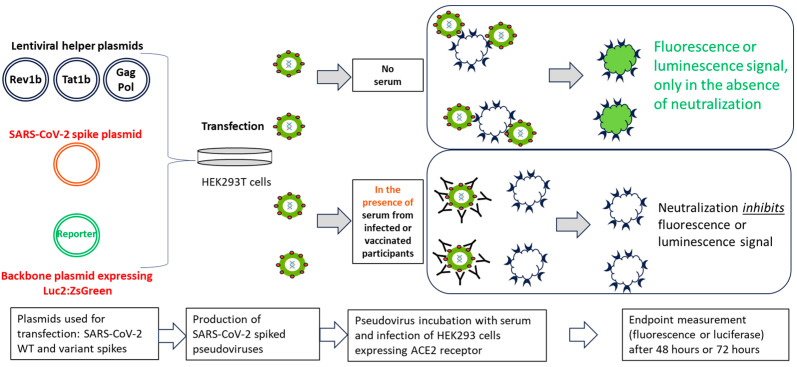
Schematic illustration of the PNT. Lentiviral helper plasmids (encoding Rev1b, Tat1b, Gag, and Pol proteins, lacking the gene for the envelope protein), a SARS-CoV-2 ancestral or variant S protein plasmid, and reporter genes (*luc2* and *ZsGreen*) were cotransfected into HEK293T cells (adapted from Crawford, et al.) [13]. Transfected cell culture supernatants were used to infect 293T/hACE2 cells in the presence or absence of serum from infected or vaccinated participants, followed by measurement of the reporter protein expression (either luminescence or fluorescence). Neutralization of pseudovirus expressing SARS-CoV-2 S protein by the anti-S antibodies inhibits the expression of the reporter protein; therefore, the RLU reading in the presence of the test serum is inversely proportional to the neutralization index of that serum. ACE2, angiotensin-converting enzyme 2; HEK, human embryonic kidney; PNT, pseudovirus neutralization assay; SARS-CoV-2, severe acute respiratory syndrome coronavirus 2; WT, wild type.

A 96-well plate format pseudovirus-based assay was developed to assess the neutralization antibody titers for the ancestral or variant (Omicron BA.1, BA.5, XBB.1.5, XBB.1.16, and XBB.2.3) strains. The PNT was conducted as shown in Figure 1. Pseudoviruses expressing the S protein of the ancestral and variant strains were used. Serum from convalescent participants infected with SARS-CoV-2 or participants vaccinated with NVX-CoV2373 as part of a clinical trial was heat-inactivated at 56 °C for 30 min, followed by serial dilution. The serially diluted serum (50 µL) was mixed with an equal volume of pseudovirus and incubated at 37 °C for 2 h. The serum and virus mixtures were then used to infect the 293T/hACE2 cells and incubated at 37 °C for 48 h to 72 h, followed by measuring the luminescence as the assay endpoint using Bright-Glo^TM^ luciferase reagent and a SpectraMax^®^ iD3 plate reader (Molecular Devices, San Jose, CA, USA). There were two controls used on each plate—a virus control (pseudoviruses incubated with cells without serum) and cell control (cells only, without pseudoviruses and serum). Additionally, sera with different neutralization levels (e.g., high, medium, and low) were established as quality control samples in each run as part of the assay system suitability.

A reduction in the luminescence expressed as relative luminescence units (RLU) in the presence of the serum indicated the neutralization of the virus infection. The 50% inhibitory titer was calculated using a 4-parameter logistical fit curve and was compared with the virus control after subtracting the cell control background. The reciprocal dilution at which the serum inhibited pseudovirus infection by 50% based on the background-adjusted RLU was reported as the 50% neutralization titer of the serum sample (ID_50_).

### 2.4. Serum Samples

Commercially available serum samples from convalescent human participants and healthy human serum samples collected in 2018 before the COVID-19 pandemic were obtained from BioIVT (Westbury, NY, USA). Human convalescent serum samples collected during the COVID-19 pandemic or from participants enrolled in the 2019nCoV-101 (NCT04368988) trial and vaccinated with NVX-CoV2373 were from the Novavax clinical sample repository (Novavax, Gaithersburg, MD, USA). The participants providing sera had consented to the use of their sera for analytical development purposes. Positive COVID-19–convalescent serum samples known to have high, medium, and low pseudovirus inhibition titers were used as the testing serum samples.

### 2.5. PNT Evaluation

Assay performance was evaluated according to precision and linearity. Precision was defined as the closeness of the measurements observed when testing the same set of samples multiple times in the same assay run (intra-assay) and in different assay runs (inter-assay) by different operators. To determine the assay precision, the serum samples were tested by two operators (analysts) on two different days in duplicate. For the ancestral strain, high-, medium-, and low-titer samples were used as quality controls. For variants, two or three positive samples were used.

Linearity evaluated the ability of a method to obtain results (e.g., neutralizing titers) that were directly proportional to the amount of analyte (e.g., neutralizing antibodies in a sample). One sample was serially diluted (6 times, 4-fold dilution series) in negative human serum and evaluated in duplicate by two different operators. The observed neutralization titer was compared to the expected neutralizing titer for each dilution.

### 2.6. Correlation Analyses

For correlation with a live wild-type virus–based microneutralization (MN) assay, the samples were tested using a validated live virus MN assay (360biolabs, Melbourne, Australia) and using a previously published method [14], followed by comparison of the data with the PNT results using linear regression analysis.

For correlation with anti-S IgG antibodies and hACE2 binding inhibition, the samples were tested using a validated anti-S IgG assay (Clinical Immunology, Novavax) and using a previously published method [15] or a validated hACE2 binding inhibition assay (Clinical Immunology, Novavax) [16], followed by comparison of the data with the PNT results using linear regression analysis.

### 2.7. Clinical Utility

To assess clinical utility, the clinical serum samples from participants in the 2019nCoV-101 NVX-CoV2373 vaccine trial were tested against ancestral and variant (Omicron BA.1, BA.2, and BA.5) strains using the PNT. The neutralizing antibody responses were profiled. To demonstrate the effects of vaccine boosters on the neutralizing antibody responses in the PNT, a matched pair of serum samples (pre and post booster doses of NVX-CoV2373) from one participant was tested in the PNT and shown as an example.

## 3. Results

### 3.1. Assay Development/Optimization

**Figure 2:**
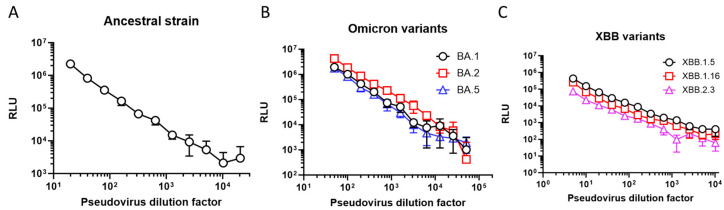
PNT optimization: luciferase endpoint. RLU of 293T/hACE2 cells infected with different amounts of pseudoviruses (as represented by viral dilution factors on the *X*-axes) expressing either the S protein of SARS-CoV-2 (**A**) ancestral strain, (**B**) BA variants, or (**C**) XBB variants. RLU was measured 2 days (ancestral and Omicron variants) or 3 days (XBB variants) post infection. hACE2, human angiotensin-converting enzyme 2; RLU, relative luminescence units.

As a first step in developing the PNT, the virus was titrated. Various 2-fold dilutions of SARS-CoV-2 ancestral (starting dilution of 1:20), Omicron BA.1, BA.2, and BA.5 (starting dilution of 1:50), and XBB.1.5/1.16/2.3 (at starting dilution of 1:5) strains were performed, followed by infection of the 293T/hACE2 cells. The RLUs for each set of viruses at 2 days post infection were plotted against the virus dilution factor and are shown in Figure 2. The RLU was the highest (2.2 × 10^6^) at the 1:20 dilution for the ancestral strain and showed a dose-proportionate signal from dilutions of 1:20 to 1:10,240. Similarly, the highest reading for the Omicron BA.1, BA.2, and BA.5 strains was observed at the 1:50 dilution, ranging from 1.7 × 10^6^ to 4.2 × 10^6^ RLU with a dose-proportionate signal from 1:50 to 1:51,200. XBB.1.5, XBB.1.16, and XBB.2.3 showed slightly lower signals (74,023 to 437,152 RLU) even at the lowest dilutions tested (1:5), and dose-proportional linearity was observed between 1:5 and 1:2560. Dose-dependent luminescence levels with decreasing amounts of pseudoviruses used for infection were observed. The RLU endpoint in these experiments also showed a robust dynamic range (>2 log range in the signal between the minimum and maximum dilutions of the pseudovirus) and user-friendliness (ease of use and throughput).

**Figure 3:**
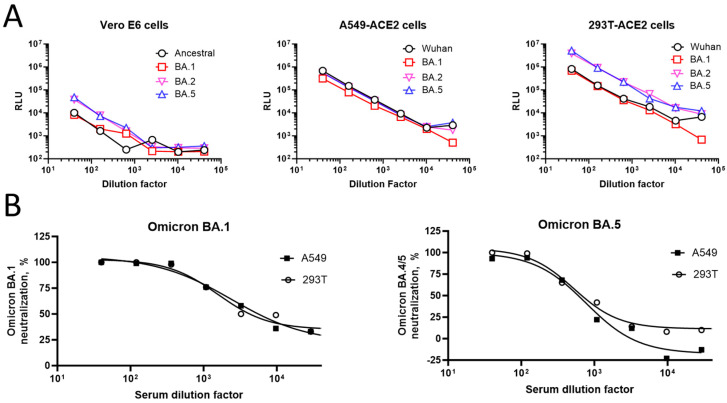
Suitability of cell lines for pseudovirus infections. (**A**) Three different cell lines (Vero-E6 (**left**), A549/hACE2-TMPRSS2 [overexpressing hACE2 and TMPRSS2] (**middle**), and 293T/hACE2 (**right**)) were infected with various amounts of pseudoviruses expressing either ancestral or variant (Omicron BA.1, BA.2, and BA.5) strain S proteins. RLU levels in the infected cells after 2 days are shown on the *Y*-axis. (**B**) Luminescence measurement of 293T/hACE2 and A549/hACE2-TMPRSS2 cells infected with Omicron BA.1 (**left**) or BA.5 (**right**) pseudoviruses in the presence or absence of increasing dilutions of serum (#2127). Cells infected with the pseudoviruses in the absence of test serum #2127 or cultured with no pseudoviruses were used as virus and cell controls, respectively. Percent neutralization compared to the virus control is shown on the *Y*-axis. hACE2, human angiotensin-converting enzyme 2; RLU, relative luminescence units; TMPRSS2, transmembrane serine protease 2.

To further identify suitable cells for pseudovirus infection, three different cell lines (Vero-E6, A549/hACE2-TMPRSS2, and 293T/hACE2; same cell numbers/well for each cell line) were compared using the pseudoviruses expressing the ancestral, Omicron BA.1, BA.2, and BA.5 S proteins (Figure 3). In the Vero-E6 cells, the RLU ranged from 206 to 47,813 for different pseudoviruses from the highest (1:2560) to the lowest (1:40) dilution. The luminescence signal plateaued at dilutions beyond 1:2560. By comparison, in the A549/hACE2-TMPRSS2 cells, the luminescence signal ranged from 508 to 687,705 for different pseudoviruses from the highest (1:40,960) to the lowest (1:40) dilution, demonstrating >1 log-higher signals compared to the Vero-E6 cells. In the 293T/hACE2 cells, higher signals were observed for the Omicron BA.2 and BA.5 strains (3.9 × 10^6^ and 5.2 × 10^6^ RLU, respectively), compared to the ancestral and BA.1 strains (8.2 × 10^5^ and 6.9 × 10^5^ RLU, respectively) at the lowest dilution tested. This 1-log difference in the signal was consistently observed for the subsequent dilutions.

The percent neutralization of the Omicron BA.1 and BA.5 pseudoviruses by various dilutions of sample serum #2127 relative to the virus control is shown in Figure 3B. In the neutralization assay, both the 293T/hACE2 and A549/ACE2-TMPRSS2 cells had similar ID_50_ values across strains.

Quantification of the luminescence signals according to TCID_50_/mL was conducted to confirm whether there was differential utilization of the cellular ACE2 or TMPRSS2 receptors by the pseudoviruses of different strains (Table 1).

The suitability of the cell lines for the PNT was further confirmed by infecting the 293T/hACE2 and A549/ACE2-TMPRSS2 cells with the Omicron BA.1 or BA.5 pseudoviruses in the presence or absence of various dilutions of the test serum samples (Table 2), followed by measurement of the luminescence.

With the suggested differential susceptibility of the cells to pseudovirus infection (reduced for the Vero-E6 cells) and the higher levels of luminescence in the 293T/hACE2 and A549/hACE2-TMPRSS2 cells, the latter cell lines were considered suitable for use in the PNT and used for further experiments.

**Figure 4:**
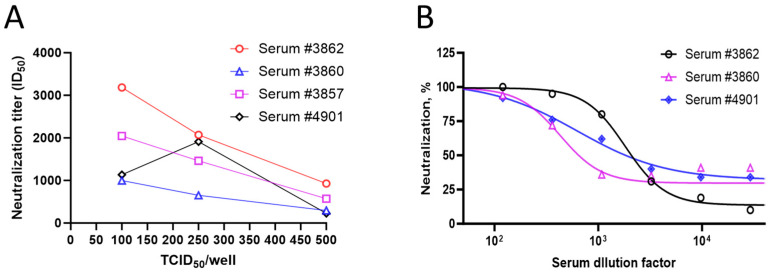
Pseudovirus dose dependence and kinetics of endpoint (luminescence). (**A**) Four test serum samples were evaluated in the PNT, with increasing amounts of the ancestral strain pseudovirus (100, 250, and 500 TCID_50_/well). ID_50_ values of each serum are shown on the *Y*-axis. (**B**) Percent neutralization of serum dilutions with 250 TCID_50_/well of ancestral strain pseudovirus at each dilution level.

To evaluate the dose dependence of the virus inoculum on the serum neutralization titers, four serum samples were tested using three different amounts (100, 250, and 500 TCID_50_/well) of pseudovirus expressing the ancestral S protein with the 293T/hACE2 cells. Three out of four samples showed the dose dependence of the pseudovirus-based neutralization titer (PNT ID_50_). A lower neutralization index (ID_50_) of the serum samples was observed with increasing amounts of the virus (TCID_50_/well from 100 to 500) (Figure 4A), suggestive of the dose dependence of the pseudovirus used. These results are in line with the stoichiometry, as higher amounts of the S protein require more antibodies to neutralize the viral infection. However, as the goal of our assay optimization was to establish a sensitive assay without compromising the assay variability, a medium virus titer of 250 TCID_50_/well was chosen for the next set of experiments. Serum samples were evaluated in the neutralization assay with 250 TCID_50_/well of the virus. A dose-dependent decrease in the percent neutralization was observed with increasing dilutions of the serum (Figure 4B), suggesting the suitability of 250 TCID_50_/well of virus inoculum for neutralization experiments.

To optimize the assay duration, 48 h and 72 h timepoints for luminescence were compared using the ancestral and Omicron BA.1 strains as examples at doses ranging from 50 to 3.13 µL/well (Table 3). The kinetics of the PNT endpoint were evaluated using the luminescence assay and by comparing the signal (i.e., RLU)-to-background (S/B) ratios at each timepoint. The S/B was higher at 48 h than at 72 h for all strains and virus amounts; therefore, 48 h detection was chosen for the pseudovirus-based neutralization assay.

### 3.2. Assay Quality

**Figure 5:**
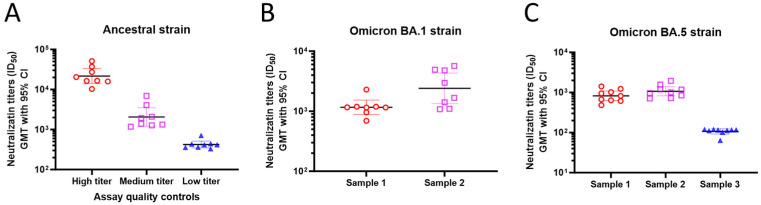
PNT precision. Human serum samples were evaluated in the PNT on different days. Duplicate samples were also tested in the same assay to evaluate intra-assay precision. Neutralization titers (ID_50_, GMT, 95% CI) are plotted on the *Y*-axis for the (**A**) ancestral, (**B**) Omicron BA.1, and (**C**) Omicron BA.5 strains. GMT, geometric mean titer; PNT, pseudovirus neutralization assay.

Consistent assay performance is needed for reliable measurement of the neutralization index of the test serum samples. To address the assay quality, the precision was evaluated for the ancestral, Omicron BA.1, and BA.5 variant strains within the same assay run and different assay runs using different titer levels of the serum samples. For the ancestral strain, high-, medium-, and low-titer serum samples were tested (Figure 5A). For Omicron BA.1 and BA.5, respectively, two to three serum samples were tested (Figure 5B,C). These samples showed consistent neutralization titers (ID_50_) within and between assays, similar to what was observed with the ancestral strain.

**Figure 6:**
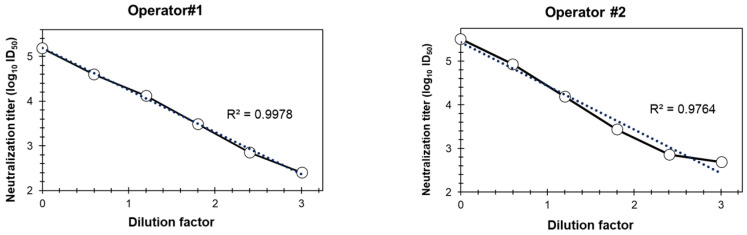
Linearity of the PNT. A test serum (#2127) was serially diluted (6 times, 4-fold dilution series), followed by evaluation with the ancestral strain, in duplicate at each dilution and by two operators, as described in Methods. Neutralization titers (log_10_ ID_50_) of the different dilutions are plotted on *Y*-axis. PNT, pseudovirus neutralization assay. Dotted line is the trend line and solid line is connectivity between the actual data points.

As part of the assay quality evaluation, linearity was evaluated in the ancestral strain–based pseudovirus assay by two operators by serially diluting serum #2127 and measuring the neutralization titers (ID_50_) (Figure 6). Dose-proportional neutralization with serum dilutions by different operators confirmed the dose-dependent decrease in the neutralization titers with serial dilutions; the *R*^2^ values are 0.9978 and 0.9764 for the two operators, respectively.

### 3.3. Correlation with Other Markers

**Figure 7:**
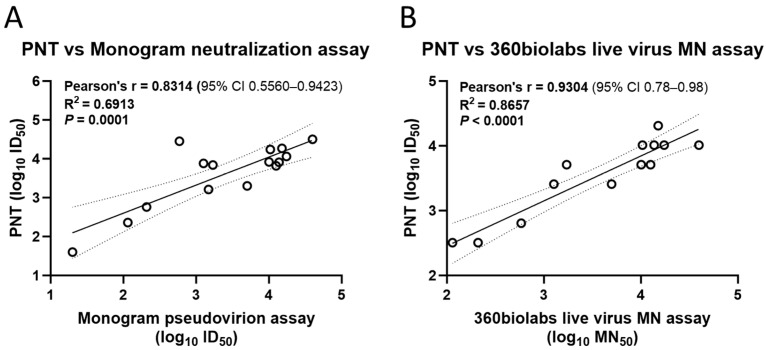
Correlation of the PNT with validated neutralization assays. Test serum samples from a clinical study were evaluated against the ancestral strain as per the method mentioned above, followed by regression analysis with a (**A**) validated neutralization assay and (**B**) live virus microneutralization assay (n = 13). Analysis was performed using GraphPad Prism^®^ software (9.3.1). Dotted line depicts the 95% CI. Solid line is Pearson’s correlation line. MN, microneutralization; PNT, pseudovirus neutralization assay.

To evaluate the concordance of the PNT results with other assays, correlation analysis was performed. The results from the PNT for the ancestral strain significantly correlated with a validated neutralization assay (Pearson’s *r* = 0.8314, *R*^2^ = 0.6913, *p* = 0.0001) (Figure 7A) and a live-virus MN assay (Pearson’s *r* = 0.9304, *R*^2^ = 0.8657, *p* < 0.0001) (Figure 7B).

**Figure 8:**
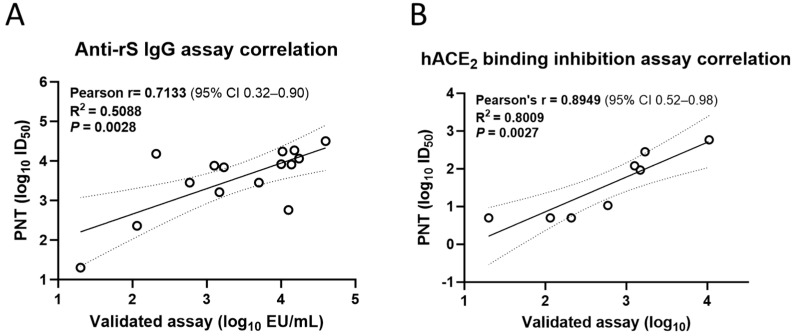
Correlation of the PNT with validated immunological assays. Test serum samples from a clinical study were evaluated in the PNT against the ancestral strain as per the method mentioned above, followed by regression analysis with a (**A**) validated anti-rS IgG assay (n = 15) and (**B**) a validated hACE2 binding inhibition assay (n = 8) Analysis was performed using GraphPad Prism^®^ software (9.3.1). Dotted line shows 95% CI. Solid line is Pearson’s correlation line. hACE2, human angiotensin-converting enzyme 2; IgG, immunoglobulin G; PNT, pseudovirus neutralization assay; rS, recombinant spike; S, spike.

Similarly, the anti-recombinant S (rS) IgG (Pearson’s *r* = 0.7133, *R*^2^ = 0.5088, *p* = 0.0028; Figure 8A) and hACE2 binding inhibition (Pearson’s *r* = 0.8949, *R*^2^ = 0.8009, *p* = 0.0027; Figure 8B) titers from the PNT for the ancestral strain significantly correlated with those from the validated assays.

### 3.4. Clinical Utility

**Figure 9:**
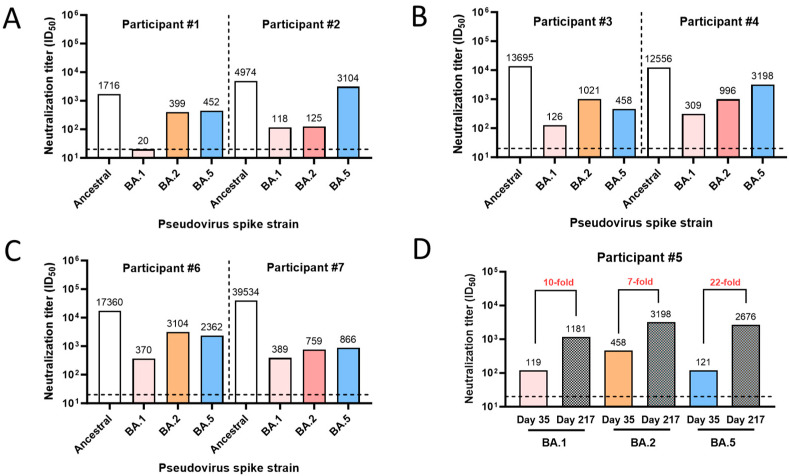
Clinical utility of the PNT. (**A**–**C**) Serum samples were used from participants #1 to 4, 6, and 7 from a clinical study where they were vaccinated with NVX-CoV2373. Samples were tested in the PNT against the ancestral or a variant (Omicron BA.1, BA.2, and BA.5) strain. (**D**) Neutralization titers (ID_50_) for serum from participant #5, collected on day 35 (14 days after primary series vaccination with NVX-CoV2373) and day 217 (28 days after a booster dose). The horizontal dashed line represents the limit of detection. PNT, pseudovirus neutralization assay.

The PNT was also used to quantify and profile the neutralization activity in the clinical serum samples. To evaluate the utility of the PNT, the serum samples were profiled for their neutralization activity against the ancestral and variant (Omicron BA.1, BA.2, and BA.5) strains (Figure 9). Higher neutralization activity was observed against the ancestral strain but with varying degrees of neutralization across other variants for participants 1 to 4 and 6 to 7 (Figure 9A–C). To investigate the effect of booster vaccination on the ancestral strain or these variants, paired serum samples from participant #5 after the primary series (day 35, 14 days after the primary series) and the first booster dose (day 217, 28 days after the 1st booster) were subjected to the PNT. A 7- to 22-fold increase in the PNT titers against the Omicron BA strains tested was observed after the booster dose (Figure 9D). These results demonstrate differences in neutralization activity among the strains and the potential clinical utility of the PNT.

### 3.5. Neutralization Titers in Convalescent Human Serum Samples against XBB Variants

**Figure 10:**
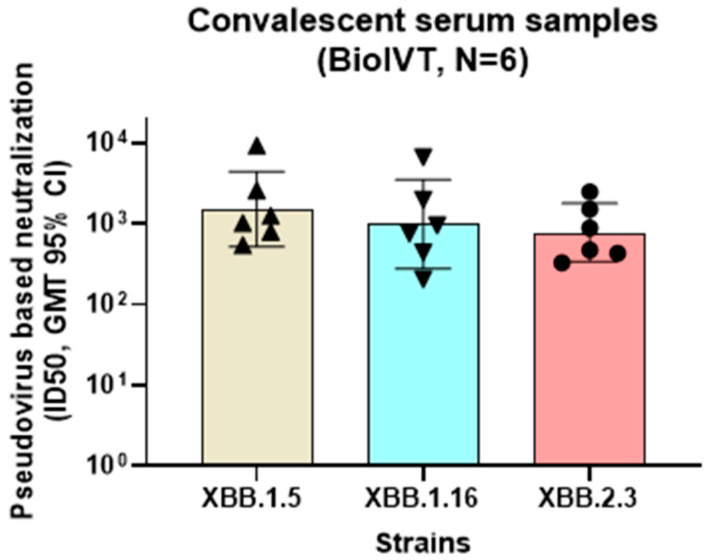
Neutralization activity of convalescent serum samples against Omicron XBB strains. Convalescent serum samples (BioIVT) were evaluated for neutralization activity in the PNT for XBB strains (XBB.1.5, XBB.1.16, and XBB.2.3). Serum samples showing >500 titer ID_50_ (n = 6) against XBB.1.5 were compared for neutralization activity against XBB.1.16 and XBB.2.3. No significant differences were observed (ordinary one-way ANOVA analysis, multiple comparisons (*p* > 0.5). GMT, geometric mean titer; PNT, pseudovirus neutralization assay.

Using a similar method as described above, a PNT was also developed against each of the XBB.1.5, XBB.1.16, and XBB.2.3 strains to aid in the characterization of an XBB.1.5 strain–based vaccine booster. The convalescent human serum samples were acquired from a commercial source, followed by evaluation in PNT assays of the XBB strains (Figure 10). The neutralization titers of the serum samples were comparable across the XBB.1.5, XBB.1.16, and XBB.2.3 strains (GMTs of 1522, 984.8, and 780, respectively) in the convalescent participants, suggesting potential cross-neutralization between XBB strains.

## 4. Discussion

This article describes the development of a pseudovirus-based neutralization assay for evaluating the neutralizing antibodies against emerging SARS-CoV-2 variants in cost-effective BSL-2 research conditions. A dose-dependent response to the pseudovirus-based neutralization titer was observed, with an acceptable assay precision across the ancestral strain and variant strains (Omicron BA.1 and BA.5). Assay linearity was demonstrated for the ancestral strain–based pseudovirus assay. The results from the PNT significantly correlated with the results from a validated live virus–based MN assay, anti-rS IgG assay, and hACE2 binding inhibition assay.

The clinical utility of this assay was demonstrated with clinical serum samples using both the ancestral strain and a series of Omicron variants (BA.1, BA.2, BA.5, XBB.1.5, XBB.1.16, and XBB.2.3). The results indicated increased neutralization activity against Omicron variants in the post booster serum compared with the activity observed after primary series vaccination. Importantly, repeat boosting seems to enhance the breadth of the neutralization responses across variants, suggesting the presence of conserved epitopes, potentially including the receptor-binding domain [17] and those that may be recognized by broadly neutralizing antibodies [18].

Assay duration is an important parameter in development and optimization. A longer assay duration might negatively affect the setup and turnaround frequency/week. The plaque reduction neutralization test (PRNT) is conventionally used for SARS-CoV-2 neutralizing antibody detection; however, it requires a BSL-3 facility and several days to obtain results, which can increase the assay cost [19,20]. The optimal signal with the PNT occurred at 48 h versus 72 h. By contrast, a shorter duration may reduce the assay sensitivity. Enzyme-linked immunoabsorbance assays (ELISAs) can be performed on a large number of samples in a conventional laboratory to measure binding antibodies in 1 day; however, they lack the ability to assess the neutralizing function of virus-specific antibodies [19,20].

The Vero-E6, A549/hACE2-TMPRSS2, and 293T/hACE2 cell lines had differential susceptibility to infection with pseudoviruses expressing S proteins from different SARS-CoV-2 strains. The Vero-E6 cells showed lower pseudovirus infection (lower TCID_50_), regardless of the virus strain, potentially because of the normal (not overexpressed) levels of ACE2 and TMPRSS2 compared to the other two cell lines. For the ancestral strain, the infectivity was similar for the A549/ACE2-TMPRSS2 and 293T/ACE2 cells, but the infectivity was higher in the t293T/ACE2 cells for the Omicron-based pseudoviruses.

A recent paper using phase 3 data on NVX-CoV2373 demonstrated that pseudovirus neutralization antibody concentrations can serve as a correlate of protection for the NVX-CoV2373 vaccine’s efficacy [3]. This paper also reported that higher neutralizing antibody titers trended with increased vaccine efficacy; however, it is important to note that while measurement of these titers is highly informative, they do not represent the complete immune response, such as cellular immunity. The results described here with the PNT show a significant correlation with a validated anti-rS total IgG assay, a validated MN assay based upon the live wild-type virus, and a hACE2 receptor binding inhibition assay, indicating that this pseudovirus-based neutralization assay could potentially be used as a surrogate marker for vaccine-induced protection against COVID-19.

Our results also demonstrate that the PNT was useful for the potentially immune-escaping Omicron and XBB variants detected in the clinical samples and for measuring the neutralizing antibody responses in the convalescent human serum samples, suggesting the utility of this assay for immunoprofiling and to assess the neutralization responses to novel variants as they emerge.

A limitation of the PNT is the need for specialty reagents such as the pseudovirus packaging system, a mammalian expression plasmid with SARS-CoV-2 S protein cDNA, and a permissive cell line. A general limitation to pseudovirus assays is that while they mimic the process of receptor interaction and cell entry, they do not support productive infection of a live infectious virus [11]. This limitation underscores the importance of validating pseudovirus-based assays against live infectious virus assays, such as the MN assay described here. Finally, the use of plasmids expressing unique S proteins allows for the investigation of prototype and variant viral strains in a pseudovirus system; however, the distribution/density of these proteins on pseudovirions may not be a direct reflection of the expression levels and epitopes occurring on natural virions.

The advantages of the PNT are that it is a BSL-2–based, high-throughput, rapid, and cost-effective alternative for a live infectious virus-based neutralization assay. The adaptability of the PNT is an important feature to accommodate the changes in the S protein among viral variants [5]. Currently, a PNT against Omicron XBB.1.5 and XBB.1.16 and other emerging variants is being developed, and sera from the NVX-CoV2373 clinical trials are being evaluated in the assay. Other future work could include using the PNT to assess the duration of the neutralization response and the correlates of protection from severe disease.

## 5. Conclusions

The PNT described here is a high-throughput, rapid, cost-effective option for the ancestral strain and the Omicron BA.5 and XBB.1.5 variants. The PNT results correlated significantly with the results from an anti-rS IgG assay and other neutralization assays (such as the MN assay). This assay will be useful for profiling the neutralizing antibody levels in clinical serum samples and will further enable the development of effective vaccines against emerging variants of SARS-CoV-2. Future work will be focused on adapting the PNT assay to emerging SARS-CoV-2 variants and beyond.

## Figures and Tables

**Table 1:** Quantitation of pseudovirus infectivity (TCID_50_/mL) in different cell lines.

Cell Line	TCID_50_/mL			
	Ancestral	BA.1	BA.2	BA.5
Vero-E6	20,380	17,820	46,240	53,120
A549/hACE2-TMPRSS2	498,600	398,480	367,420	411,280
293T/hACE2	543,700	663,660	1,249,340	834,400

hACE2, human angiotensin-converting enzyme 2; TMPRSS2, transmembrane serine protease 2.

**Table 2:** Evaluation of human serum samples (COVID-19–convalescent or from NVX-CoV2373–vaccinated trial participants) for pseudovirus neutralization (ID_50_) in 293T/hACE2 and A549/hACE2-TMPRSS2 cells.

Sample ID	PNT ID_50_ BA.1 Titer		PNT ID_50_ BA.5 Titer	
	293T	A549	293T	A549
Serum #8522	<40	<40	NT	NT
Serum #2127	4223	2905	674	603
Serum #2124	4782	5811	1950	1524
Serum #7781	<40	<40	40	43
Serum #8061	NT	NT	102	126

hACE2, angiotensin-converting enzyme 2; NT, not tested; PNT, pseudovirus neutralization assay; TMPRSS2, transmembrane serine protease 2.

**Table 3:** Optimization of assay incubation time duration (S/B ratio endpoint).

Virus/Well (µL)	Ancestral		BA.1	
	48 h	72 h	48 h	72 h
50	735	329	636	427
25	339	161	224	147
12.5	152	39	65	72
6.25	97	32	32	34
3.13	66	15	18	18

h, hour; S/B, signal-to-background ratio.

## Data Availability

Requests for the data presented in this study will be considered by the corresponding author. The data are not publicly available because of proprietary subject and sample information.
